# Polymorphisms Related to the Serum 25-Hydroxyvitamin D Level and Risk of Myocardial Infarction, Diabetes, Cancer and Mortality. The Tromsø Study

**DOI:** 10.1371/journal.pone.0037295

**Published:** 2012-05-23

**Authors:** Rolf Jorde, Henrik Schirmer, Tom Wilsgaard, Ragnar Martin Joakimsen, Ellisiv Bøgeberg Mathiesen, Inger Njølstad, Maja-Lisa Løchen, Yngve Figenschau, Jens Petter Berg, Johan Svartberg, Guri Grimnes

**Affiliations:** 1 Tromsø Endocrine Research Group, Department of Clinical Medicine, University of Tromsø, Tromsø, Norway and Division of Internal Medicine, University Hospital of North Norway, Tromsø, Norway; 2 Department of Clinical Medicine, University of Tromsø, Tromsø, Norway; 3 Department of Community Medicine, University of Tromsø, Tromsø, Norway; 4 Brain and Circulation Research Group, Department of Clinical Medicine, University of Tromsø, Tromsø, Norway and Department of Neurology and Neurophysiology, University Hospital of North Norway, Tromsø, Norway; 5 Department of Medical Biology, Faculty of Health Sciences, University of Tromsø, Tromsø, Norway and Department of Laboratory Medicine, University Hospital of North Norway, Tromsø, Norway; 6 Department of Medical Biochemistry, Institute of Clinical Medicine, University of Oslo and Oslo University Hospital, Oslo, Norway; Peninsula College of Medicine and Dentistry, United Kingdom

## Abstract

**Objective:**

Low serum 25(OH)D levels are associated with cardiovascular risk factors, and also predict future myocardial infarction (MI), type 2 diabetes (T2DM), cancer and all-cause mortality. Recently several single nucleotide polymorphisms (SNPs) associated with serum 25-hydroxyvitamin D (25(OH)D) level have been identified. If these relations are causal one would expect a similar association between these SNPs and health.

**Methods:**

DNA was prepared from subjects who participated in the fourth survey of the Tromsø Study in 1994–1995 and who were registered with the endpoints MI, T2DM, cancer or death as well as a randomly selected control group. The endpoint registers were complete up to 2007–2010. Genotyping was performed for 17 SNPs related to the serum 25(OH)D level.

**Results:**

A total of 9528 subjects were selected for genetic analyses which were successfully performed for at least one SNP in 9471 subjects. Among these, 2025 were registered with MI, 1092 with T2DM, 2924 with cancer and 3828 had died. The mean differences in serum 25(OH)D levels between SNP genotypes with the lowest and highest serum 25(OH)D levels varied from 0.1 to 7.8 nmol/L. A genotype score based on weighted risk alleles regarding low serum 25(OH)D levels was established. There was no consistent association between the genotype score or individuals SNPs and MI, T2DM, cancer, mortality or risk factors for disease. However, for rs6013897 genotypes (located at the 24-hydroxylase gene (*CYP24A1*)) there was a significant association with breast cancer (P<0.05).

**Conclusion:**

Our results do not support nor exclude a causal relationship between serum 25(OH)D levels and MI, T2DM, cancer or mortality, and our observation on breast cancer needs confirmation. Further genetic studies are warranted, particularly in populations with vitamin D deficiency.

**Trial Registration:**

ClinicalTrials.gov NCT01395303

## Introduction

Vitamin D is an ancient hormone with indisputable importance in calcium and bone metabolism. The vitamin D receptor (VDR) is a nuclear receptor localised in cells in a number of organs indicating important functions also in extra-skeletal tissues [1].

Thus, in previous reports from the Tromsø Study we have found low serum 25-hydroxyvitamin D (25(OH)D) levels (which is the vitamin D metabolite used to evaluate a subject's vitamin D status) to be strongly related to hypertension [2], obesity [3], elevated glycated haemoglobin (HbA_1c_) [4] and an unfavourable serum lipid profile [5]. In line with this we have found low serum 25(OH)D levels to be associated with the risk of developing type 2 diabetes mellitus (T2DM) [6] as well as with increased all-cause mortality [7]. Similar observations have been reported from other studies [8], [9], and the serum 25(OH)D levels have also been associated with cancer [10]. Accordingly, the serum 25(OH)D level appears to be an important risk factor for disease in general. However, whether there is a causal relation between low serum 25(OH)D and disease is uncertain and awaits the results of properly performed randomized clinical trials (RCT).

Intake of vitamin D and sun exposure are major determinants of the serum 25(OH)D level, but cannot fully explain differences in serum 25(OH)D between individuals [11]. Based on family and twin studies it has been estimated that more than 50% of the variability can be ascribed to genetic factors [12], and recently several single nucleotide polymorphisms (SNPs) related to serum 25(OH)D have been identified [13], [14]. These SNPs, where the minor allele frequencies vary between 16 and 40%, appear as important for the serum 25(OH)D level as the effect of vitamin D supplementation and nearly as important as the effect of season [13]. Accordingly, if the serum 25(OH)D level is causally related to health, one would expect that subjects with polymorphisms associated with low serum 25(OH)D levels would be more prone to disease than subjects with presumably more favourable polymorphisms.

The Tromsø Study is a longitudinal epidemiological population health study with repeated surveys conducted at 6–7 years intervals [15]. In the fourth survey performed in 1994–1995, blood samples for preparation of DNA were collected in close to 27 000 subjects. The participants are followed-up with registration of incident myocardial infarction (MI), T2DM, cancer and death, and we therefore had the opportunity to test if SNPs in the vitamin D system are related to hard endpoints as well as cardiovascular disease (CVD) risk factors. However, the study did not provide consistent evidence for such relations.

## Materials and Methods

### The Tromsø study

The Tromsø Study, conducted by the University of Tromsø in cooperation with the National Health Screening Service, is a longitudinal population-based multipurpose study focusing on lifestyle related diseases. The fourth survey was performed in 1994–1995, the fifth in 2001–2002 and the sixth in 2007–2008; 27 158, 8130 and 12984 subjects attended, respectively (Table 1) [15], [16].

**Table 1:** Event flow in the Tromsø study relevant for the present study.

Time point	Event in Tromsø study	Subjects (n)	Relevant examinations	Included in present SNP study (n)
1994–1995	4^th^ survey first visit	27 158	Health questionnaire	9471
			Physical examination	9471
			Blood clot	9471
			Serum lipids	9471
	4^th^ survey second visit	7965	HbA_1c_	4613
			Serum 25(OH)D	4567
2001–2002	5^th^ survey	8130	Serum PTH	3809
2007–2008	6^th^ survey	12 984		
31. Dec 2007	Cut off point for MI			2025
31. Dec 2008	Cut off point for T2DM			1092
	Cut off point for cancer			2924
31. Dec 2010	Cut off point for mortality			3828

### Definition of T2DM endpoints

Possible cases of T2DM were identified through self-reported diabetes in questionnaires in the fourth (1994–1995), fifth (2001–2002) and sixth (2007–2008) surveys of the Tromsø Study, through elevated HbA_1c_ (>6.5%) in one of the two former health surveys, by linkage of the fourth survey participant list to the University Hospital of North Norway digital discharge diagnosis registry (ICD-9 codes 250, 357.2, 362.0, 583.8, 648.0, 648.8, 790.2, ICD-10 codes E10.0–E14, O24 and R73) in conjunction with the concurrent CVD registration, or by linkage to the Causes of Death Registry. In addition, we did systematic manual and electronic searches through medical charts for diabetes (before 2001 paper, from 2001 digital records) on all participants registered with the following cardiovascular diagnose codes in the International Classification of Diseases version 9 and 10: ICD-9 codes 410–414 (ischemic heart disease), 427 (cardiac dysrhythmia), 430–438 (cerebrovascular disease), 798 (sudden death) and 799 (other ill-defined and unspecified causes of mortality), and ICD-10 codes I20–I25 (ischemic heart disease), I47 (paroxysmal tachycardia), I47 (atrial fibrillation and flutter), I60–I69 (cerebrovascular disease), R96 (other sudden death, cause unknown) and R98 (unattended death) and R99 (other ill-defined and unspecified causes of mortality). Cases of diabetes registered by means of one of these procedures were verified by medical record information at the University Hospital of North Norway, the only local hospital serving the Tromsø population. Cases of unknown type of diabetes were not included in the analyses, and only cases of verified T2DM were used. Differentiation between type 1 and type 2 diabetes mellitus was based on clinical judgement and on measurements of C-peptide and/or glutamic acid decarboxylase antibody (anti-GAD). The T2DM endpoints were included till the end of 2008 [17].

**Table 2:** Characteristics of the study population in 1994–1995.

	Entire Tromsø study cohort	Present study cohort	Control cohort	Subjects with endpoint MI	Subjects with endpoint T2DM	Subjects with endpoint cancer	Dead
N	27 158	9471	4175	2025	1092	2924	3828
Males (%)	47.4	47.3	43.3	64.8	52.0	48.1	51.6
Current smokers (%)	36.1	33.3	26.1	35.1	28.4	35.3	34.1
Age (years)	46.9±15.1	59.6±13.7	65.6±12.5	64.5±12.1	60.2±12.4	59.0±13.8	67.2±12.5
Systolic BP (mmHg)	134.8±20.5	145.1±23.8	149.2±24.8	151.7±24.4	152.2±23.5	142.9±23.4	152.5±25.4
Diastolic BP (mmHg)	78.1±12.4	83.0±13.4	83.9±13.8	86.1±13.9	86.5±13.7	82.0±13.1	85.2±14.5
BMI (kg/m^2^)	25.2±3.9	26.0±4.2	25.9±4.1	26.6±4.0	29.0±4.8	25.7±4.1	26.0±4.3
Total cholesterol (mmol/L)	6.05±1.31	6.59±1.32	6.70±1.33	6.94±1.29	6.69±1.22	6.43±1.31	6.70±1.34
HDL cholesterol (mmol/L)	1.50±0.41	1.52±0.43	1.54±0.44	1.39±0.40	1.33±0.38	1.52±0.42	1.51±0.45
Triglycerides (mmol/L)	1.55±1.05	1.70±1.09	1.68±1.07	1.99±1.20	2.39±1.55	1.61±1.01	1.75±1.09
HbA_1c_ (%)*	5.45±0.66	5.53±0.72	5.51±0.74	5.63±0.87	6.36±1.45	5.50±0.63	5.64±0.86
25(OH)D (nmol/L)*	58.9±20.1	58.4±20.1	57.4±19.8	58.6±19.9	53.4±18.5	62.8±21.1	58.2±20.6

* Measured only in those attending the second visit of the Tromsø study 1994–1995.

**Table 3:** Genotype frequency and serum 25(OH)D levels in relation to genotype in the entire cohort.

Nearest gene	SNP	Genotype	Frequency	Serum 25(OH)D (nmol/L)**			P (trend)***
		major/minor	major and				
		homyzygote	minor	Major	Heterozygote	Minor	
			homozygote*	homozygote		homozygote	
GC	rs2282679	TT/GG	0.56, 0.06	60.4±20.3	56.2±19.5	52.7±18.8	<0.001
	rs7041	CC/AA	0.30, 0.20	61.4±20.3	57.7±20.0	55.4±19.4	<0.001
	rs1155563	TT/CC	0.50, 0.08	60.2±20.1	57.1±20.0	52.9±18.5	<0.001
	rs3755967	CC/TT	0.56, 0.06	60.5±20.3	56.3±19.5	53.0±19.0	<0.001
	rs17467825	AA/GG	0.56, 0.06	60.5±20.3	56.3±19.6	52.8±18.9	<0.001
	rs2298850	GG/CC	0.58, 0.06	60.3±20.3	56.2±19.5	52.5±19.0	<0.001
	rs222020	TT/CC	0.69, 0.03	58.0±20.2	59.2±20.0	58.3±19.6	ns
CYP2R1	rs10741657	GG/AA	0.34, 0.17	57.4±20.3	58.6±19.8	59.7±20.7	0.001
	rs2060793	GG/AA	0.34, 0.17	57.3±20.2	58.6±19.7	59.6±20.7	0.001
	rs12794714	GG/AA	0.35, 0.16	58.8±20.8	58.8±19.9	56.5±19.2	0.001
	rs1562902	TT/CC	0.30, 0.21	58.3±20.0	58.2±20.1	58.9±20.3	ns
NADSYN1	rs12785878	TT/GG	0.38, 0.15	59.1±20.4	58.2±20.2	57.3±19.1	<0.05
	rs3794060	TT/CC	0.38, 0.16	59.2±20.4	58.1±20.2	57.2±19.1	<0.05
	rs3829251	GG/AA	0.54, 0.08	58.8±20.3	58.3±20.1	55.9±19.0	ns
	rs11234027	GG/AA	0.54, 0.08	58.8±20.2	58.2±20.0	56.2±19.1	ns
CYP24A1	rs6013897	TT/AA	0.59, 0.06	59.2±20.0	57.2±19.8	56.5±21.8	<0.001
	rs2762939	GG/CC	0.54, 0.07	59.0±20.5	57.8±19.4	56.7±21.0	ns

* Analyzed in the entire cohort of 9471 subjects;

** analysed in 4567 subjects;

*** P, linear trend across genotypes with gender, age, BMI, vitamin D/cod liver oil supplementation and season as covariates.

### Definition of MI endpoints

Hospitalized cases of incident MI were identified by linking the Tromsø Study participant list to the discharge diagnosis register at the University Hospital of North Norway. To identify all possible first-ever MI cases, our search strategy included the following diagnostic codes: from 1980–98 ICD 9 codes 410–414 and 798–7998; and thereafter ICD 10 codes I20–I25, and R96 and R98–99. As for diabetes, we also systematically searched medical records for MI in participants registered with ICD-9 codes 427, 430–438, and 798–799, and ICD-10 codes I47–48, I60–I69, R96 and R98–99. The hospital medical record was then retrieved for case validation. Discharge letters from hospitalizations in other hospitals were also collected when appropriate. Further, the Tromsø Study participant list was linked with the nationwide Causes of Death Registry at Statistics Norway and the death certificates were retrieved for those with an underlying or contributing diagnosis of CVD or sudden unexpected death. Relevant information was collected from additional sources such as autopsy reports and records from nursing homes, ambulance services and general practitioners. This procedure identified fatal incident cases of MI that occurred as out-of-hospital deaths, including deaths occurring outside Tromsø. Cases meeting diagnostic criteria for definite or probable fatal or non-fatal first-ever MI were classified as MI. WHO MONICA/MORGAM criteria were used in the algorithms and included clinical symptoms and signs, findings in electrocardiograms, values of cardiac biomarkers and (when applicable) autopsy reports [18]. Silent MIs as defined by ECG only were not included as cases because of difficulties in determining the exact date of the event. The MI endpoints were included till the end of 2007.

### Definition of cancer and mortality endpoints

Information on cancer incidence and cancer location was retrieved from the Cancer Registry of Norway updated till the end of 2008. Information on death was obtained from the Causes of Death Registry, and information on moving out of the Tromsø area and emigration from Norway was obtained from the Norwegian Registry of Vital Statistics updated till the end of 2010.

### Selection of study cohort and power calculation

In addition to MI, T2DM, endpoint registers for stroke, hip and radial fractures, and aortic stenosis have been created as part of the Tromsø study. When the subjects for the present study were selected in December 2010, 1874 subjects were registered with MI, 1136 with T2DM, 1150 with stroke, 754 with hip fracture, 1177 with radial fracture, 569 with aortic stenosis, 2932 with cancer and 3850 were dead. In addition, 769 subjects with a total carotid plaque area in the upper quartile in the carotid ultrasound measurement at the second visit of the fourth survey were included as cases. As all of these endpoints were of potential interest regarding genetic polymorphisms, and limited funding did not allow DNA preparation and genetic analyses of the entire Tromsø Study cohort, we decided on a case-cohort design. With this approach, the same “control cohort”, randomly selected from the entire cohort who attended the fourth survey in 1994–1995, could be used as “control group” for all the different endpoints [19].

In the power calculation for deciding the size of the control cohort, we assumed a difference in serum 25(OH)D of 20 nmol/L between those with the most favourable and unfavourable genotype [13], that this difference would result in a difference in relative risk of approximately 1.3 regarding MI [20], and that 20% of the controls had the exposed polymorphism. With these assumptions and α = 0.05 including 3655 controls and 1218 cases would give a power of 0.9 to detect a difference between genotypes for MI. We therefore decided on a control cohort of 4000 subjects, but included additional subjects in case some of the genetic analyses or the DNA extractions were unsuccessful. The registration and quality control of endpoints continued until August 2011 and the final numbers of subjects with endpoints were therefore different from when the subjects for DNA analyses were selected.

### Measurements

At the survey in 1994–1995, the participants filled in questionnaires on medical history, and lifestyle factors. Blood pressure, height and weight, serum total cholesterol and triglycerides were analyzed as previously described [15].

Sera from the second visit were stored at −70°C, and after a median storage time of 13 years, thawed in March 2008 and analyzed for 25(OH)D using an automated clinical chemistry analyser (Modular E170, Roche Diagnostics®, Mannheim, Germany) [21].

In the first visit whole blood was collected for preparation of blood clots that were later stored at the HUNT/NTNU Biobank in Levanger, Mid-Norway where the DNA was also prepared using a manual isolation method based on Clotted Lysate Preparation Protocol for 8 or 16 samples on the Autopure LS Instrument from Gentra (Gentra Systems Inc. MN, US) using reagents from Qiagen (Qiagen NV, Venlo, The Netherlands).

In the fifth survey in the Tromsø study in 2001–2002 serum parathyroid hormone (PTH) was measured conclusively as previously described [22] and included in the present study as an established marker of vitamin D's biological effect [1].

Based on available data from two genome-wide association studies [13], [14], and one comprehensive association analysis [23], 17 SNPs located at or near the following genes with importance for vitamin D metabolism and reported to be the most strongly associated with the serum 25(OH)D levels, were selected for analysis in the present study:

The vitamin D binding protein (DBP) gene (*DBP or GC*): the six top SNPs according to Wang et al. [13] (rs2282679, rs7041, rs1155563, rs3755967, rs17467825, rs2298850) and one additional promising SNP reported by Bu et al. [23] (rs222020).
The 25-hydroxylase gene (*CYP2R1*) involved in the conversion of vitamin D into 25(OH)D: the top three SNPs according to Wang et al. [13] (rs10741657, rs2060793, rs12794714 (rs1993116 probably proxy for rs2060793 and not included)), and one additional promising SNP reported by Bu et al. [23] (rs1562902).
The 7-dehydrocholesterol reductase/NAD synthetase 1 gene (*NADSYN1*) responsible for the availability of 7-dehydrocholesterol in the skin: the top two SNPs according to Wang et al. [13] (rs12785878, rs3794060) and the two top SNPs according to Ahn et al. [14] (rs3829251, rs11234027).
The 24-hydroxylase gene (*CYP24A1*) involved in the degradation of 25(OH)D: rs6013897 [13]. Rs2762939 is not related to the serum 25(OH)D level, but was still included because of a potential relation to artery calcification [24], which could be relevant in the present study.

All genotyping was performed by KBioscience (http://www.kbioscience.co.uk) using KASP (KBioScience Allele-Specific Polymorphism) SNP genotyping system. KASP is a competitive allele-specific PCR incorporating a FRET (Fluorescence Resonance Energy Transfer) quencher cassette. The KASP reporting system is comprised of the following constituent oligonucleotides:

Two allele-specific primers (one for each SNP allele). Each primer contains a unique unlabelled tail sequence at its 5′ end.
One common (reverse) primer.
Two 5′ fluor-labelled oligonucleotides, one labelled with FAM, one with HEX. These oligonucleotide sequences are designed to interact with the sequences of the tails of the allele-specific primers.
Two further oligonucleotides, with quenchers bound at their 3′ ends. These oligonucleotide sequences are complementary to those of the fluor-labelled oligonucleotides (and therefore also complementary to the tails of the allele-specific primers). These quenched oligonucleotides therefore bind their fluor-labelled complements and all fluorescent signal is quenched until required.

In the initial stage of PCR, the appropriate allele-specific primer binds to its complementary region directly upstream of the SNP (with the 3′ end of the primer positioned at the SNP nucleotide). The common reverse primer also binds and PCR proceeds, with the allele-specific primer becoming incorporated into the template. During this phase, the fluor-labelled oligonucleotides remain bound to their quencher-bound complementary oligonucleotides, and no fluorescent signal is generated.

**Table 4:** Number of subjects with specific endpoints and number of subjects in corresponding control groups and hazard ratio with 95% confidence interval in relation to genotype score quartile and genotypes in the four selected SNPs.

		MI	T2DM	Cancer	Death
Endpoint (n)		2025	1092	2924	3828
Controls (n)		3295	3785	3264	2379
Genotype score	Quartile 1*	ref	ref	ref	ref
	Quartile 2	1.10 (0.97–1.24)	1.09 (0.92–1.29)	1.04 (0.94–1.16)	1.05 (0.96–1.15)
	Quartile 3	1.09 (0.96–1.23)	1.02 (0.86–1.20)	1.02 (0.92–1.13)	1.03 (0.94–1.13)
	Quartile 4	0.93 (0.82–1.06)	1.01 (0.86–1.20)	0.96 (0.87–1.07)	1.01 (0.92–1.10)
rs2298850	Major homozygote	ref	ref	ref	ref
	Heterozygote	0.91 (0.83–1.00)	0.98 (0.86–1.12)	0.98 (0.91–1.06)	0.95 (0.89–1.02)
	Minor homozygote†	0.83 (0.68–1.01)	0.87 (0.67–1.14)	1.01 (0.86–1.18)	0.97 (0.84–1.12)
rs10741657	Major homozygote†	ref	ref	ref	ref
	Heterozygote	1.05 (0.95–1.16)	1.07 (0.94–1.22)	1.03 (0.95–1.12)	1.04 (0.97–1.12)
	Minor homozygote	1.05 (0.92–1.19)	1.00 (0.83–1.19)	1.03 (0.92–1.14)	0.98 (0.89–1.08)
rs3794060	Major homozygote	ref	ref	ref	ref
	Heterozygote	1.03 (0.94–1.14)	0.99 (0.87–1.13)	1.00 (0.92–1.08)	1.04 (0.97–1.11)
	Minor homozygote†	1.00 (0.88–1.15)	0.99 (0.82–1.18)	0.94 (0.84–1.05)	1.06 (0.96–1–17)
rs6013897	Major homozygote	ref	ref	ref	ref
	Heterozygote	1.09 (0.99–1.20)	1.07 (0.95–1.22)	1.02 (0.94–1.10)	1.05 (0.98–1.12)
	Minor homozygote†	1.11 (0.92–1.34)	1.13 (0.88–1.47)	1.20 (1.03–1.41)	1.05 (0.92–1.21)

Cox regression with adjustment for age, gender, BMI and vitamin D/cod liver oil supplementation. None of the relations for the individual SNPs remained statistically significant after correction for multiple comparisons with a factor of 32.

* Quartile with highest serum 25(OH)D level.

† Risk allele.

As PCR proceeds, one of the fluor-labelled oligonucleotides corresponding to the amplified allele also gets incorporated into the template and is hence no longer bound to its quencher-bound complement. As the fluor is no longer quenched, the appropriate fluorescent signal is generated and detected by the usual means. If the genotype at a given SNP is homozygous, only one or other of the possible fluorescent signals will be generated. If the individual is heterozygous, the result will be a mixed fluorescent signal.

 In all genotyping plates no-template controls (NTCs) are included to demonstrate that any amplification in the sample wells is due solely to the presence of the sample DNA. Two separate manual quality control checks are performed, and the data is also checked by specific software to determine that there are no incorrect call assignments, no samples too close or too far from the origin, no NTCs amplified, or any incorrect calls.

**Table 5:** Number of subjects with specific types of cancer and number of subjects in corresponding control groups and hazard ratio with 95% confidence interval in relation to genotype score quartile and genotypes in the four selected SNPs.

		Breast cancer	Lung cancer	Colorectal cancer	Prostate cancer
Endpoint (n)		403	312	438	375
Controls (n)		2273	4066	3996	1665
Genotype score	Quartile 1**	ref	ref	ref	ref
	Quartile 2	1.14 (0.85–1.52)	0.89 (0.65–1.23)	0.92 (0.70–1.22)	1.11 (0.84–1.48)
	Quartile 3	1.18 (0.89–1.56)	0.99 (0.73–1.35)	1.13 (0.87–1.47)	1.15 (0.86–1.53)
	Quartile 4	1.11 (0.83–1.47)	0.87 (0.64–1.18)	1.06 (0.82–1.38)	0.99 (0.75–1.32)
rs2298850	Major homozygote	ref	ref	ref	ref
	Heterozygote	1.01 (0.82–1.24)	0.92 (0.72–1.16)	1.07 (0.88–1.31)	1.13 (0.91–1.40)
	Minor homozygote†	0.84 (0.51–1.39)	1.12 (0.71–1.77)	1.44 (1.01–2.07)	1.19 (0.80–1.78)
rs10741657	Major homozygote†	ref	ref	ref	ref
	Heterozygote	1.13 (0.91–1.41)	0.89 (0.70–1.13)	0.99 (0.80–1.22)	1.15 (0.91–1.45)
	Minor homozygote	0.96 (0.71–1.29)	0.82 (0.58–1.14)	1.02 (0.78–1.34)	1.19 (0.89–1.61)
rs3794060	Major homozygote	ref	ref	ref	ref
	Heterozygote	1.02 (0.83–1.27)	0.83 (0.65–1.05)	0.86 (0.70–1.05)	0.91 (0.73–1.14)
	Minor homozygote†	0.95 (0.71–1.29)	0.97 (0.70–1.35)	0.73 (0.54–0.99)	0.88 (0.64–1.21)
rs6013897	Major homozygote	ref	ref	ref	ref
	Heterozygote	1.22 (1.00–1.50)	0.91 (0.72–1.16)	1.05 (0.85–1.28)	0.95 (0.76–1.18)
	Minor homozygote†	1.86(1.28–2.70)*	1.02 (0.62–1.67)	1.29 (0.87–1.91)	1.10 (0.69–1.73)

* P<0.05; Cox regression with adjustment for age, gender, BMI and vitamin D/cod liver oil supplementation. Correction for multiple comparisons for the individual SNPs was performed with a factor of 32.

** Quartile with highest serum 25(OH)D level.

† Risk allele.

### Statistical analyses

The relation between SNP genotypes and the endpoints MI, T2DM, cancer and mortality were evaluated in Cox regression analyses with age, gender, body mass index (BMI), and vitamin D/cod liver oil supplementation as covariates. For MI the risk factors systolic blood pressure and serum cholesterol were included as well in a separate analysis to examine if relations could be explained through these risk factors. For mortality, the observation time was set from 1994, for the other endpoints from birth. The period of observation was cut off by the end of 2007 for MI, 2008 for diabetes and cancer, and 2010 for mortality. The predefined control cohort was used as controls for the subjects with a specific endpoint (cases). Since this control cohort was randomly selected from the entire cohort, it also included a considerable number of subjects with one or more endpoints. When analyzing a specific endpoint, subjects in the control cohort with that specific endpoint were moved to the case group (which only included subjects with that specific endpoint). Therefore, the size of the control cohort varied depending on endpoint in question. Because of the size of the control-cohort, we did not make adjustment to the partial likelihood in the Cox regression analyses [19].

Distribution of the continuous variables serum 25(OH)D, blood pressure, lipids, BMI and HbA_1c_ was evaluated for skewness and curtosis and visual inspection of histograms and found normal except for triglycerides, HbA_1c_ and PTH which were normalized by log transformation before use as dependent variables. Trends across the genotypes were evaluated with linear regression with age, gender, BMI, month of examination (with the use of dummy variables) and intake of vitamin D supplements or cod liver oil as covariates. The CVD risk factors were evaluated within the entire cohort.

The genotype frequencies were examined for compliance with Hardy-Weinberg equilibrium using chi-squared analysis [25]. Linkage disequilibrium (LD) between SNPs was evaluated with r^2^ and Lewontin's D′ statistics [26], [27].

Based on the β coefficients from regression analyses with serum 25(OH)D as dependent variable, the risk alleles (the ones associated with low serum 25(OH)D levels) were weighted and a genotype score constructed [13]. For this score only SNP significantly related to the serum 25(OH)D levels were included, and only SNPs that were uncorrelated (r^2^<0.40) [28]. In the Cox regression analyses the cohort was divided in genotype score quartiles with the lowest quartile (the one with the highest serum 25(OH)D level) as reference. In addition, the individual SNPs were also evaluated in the Cox regression with the major homozygote genotype used as reference.

The data are shown as mean ± SD. All tests were done two-sided, and a P-value<0.05 was considered statically significant. Where the individual SNPs were analyzed, corrections for multiple testing were performed with the Bonferroni method. Thus, in the Cox regression analyses the P-values were multiplied by a factor of 32 (4 SNPs and 8 endpoints) and for the relation between CVD risk factors and SNPs with a factor of 4.

### Ethics

The study was approved by the Regional Committee for Medical and Health Research Ethics (REK nord) (reference 2010/2913-4). Only participants with valid written consent were included.

## Results

A total of 9528 subjects were selected for participation in the study and DNA successfully prepared and analyzed for at least one SNP in 9471 subjects. Among these, 4175 were included as control cohort, 2025 subjects were finally registered with the endpoints MI, 1092 with T2DM, 2924 with cancer and 3828 had died (Table 1). The age distribution, gender, smoking habits and laboratory data from 1994–1995 in these subjects are shown in Table 2. The genotypes for all the SNPs were in Hardy-Weinberg equilibrium in all analyses except for the four SNPs at the *NADSYN1* gene where chi-squared testing gave P<0.05.

The individual SNP analyses were successful in 98.8–99.5% of the subjects. The serum 25(OH)D levels in relation to genotype are shown in Table 3. In general, the mean differences in serum 25(OH)D between the major and minor homozygote genotypes were for SNPs related to the *GC*, *CYP2R1*, *NADSYN1* and *CYP24A1* genes 0.3–7.8 nmol/L, 0.6–2.4 nmol/L, 1.8–2.9 nmol/L, and 2.3–2.7 nmol/L, respectively.

The SNPs with the same gene location, and that in addition were significantly related to serum 25(OH)D levels, were highly correlated. Thus, for the *GC* gene, rs2298850, which was the SNP with the highest difference in mean serum 25(OH)D between major and minor homozygotes, was in LD with rs2282679, rs1155563, rs3755967, rs17467825 (r^2^ = 0.90, 0.59, 0.92, 0.92, respectively), but not with rs7041 (r^2^ = 0.38). Similarly, for the *CYP2R1* gene, rs10741657 was in LD with rs2060793 and rs12794714 (r^2^ = 1.00 and 0.49, respectively), and for the *NADSYN1* gene rs3794060 was in LD with rs12785878 (r^2^ = 1.00). However, none of the SNPs rs2298850, rs7041, rs10741657, rs3794060 and rs6013897 was in LD with each other, and these five SNPs were used for construction of the genotype score. The mean serum 25(OH)D levels in the four quartiles of this genotype score were 62.5±20.7, 59.1±19.8, 57.3±19.5 and 54.7±19.5 nmol/L, respectively.

In the Tables 4 and 5 the results regarding the endpoints in relation to these genotype score quartiles together with the four SNPs selected because of significant and highest difference in serum 25(OH)D between major and minor homozygotes within each gene, are presented.

The genotype score was not related to serum PTH, but rs6013897 located at the *CYP24A1* gene showed a significant relation with lower mean serum PTH level for the polymorphism with the highest mean serum 25(OH)D level (Table 6).

**Table 6:** CVD risk factors in 1994–1995 and serum PTH in 2001–2002 in relation to genotype score quartile and genotypes in the four selected SNPs in the entire study cohort of 9471 subjects.

SNP and genotypes		Systolic blood pressure (mmHg)	BMI (kg/m^2^)	Total cholesterol (mmol/L)	Tri-glycerides (mmol/L)	HbA_1c_ (%)**	Serum PTH (pmol/L)***
Genotype score	Quartile 1****	145.5±24.3	26.0±4.2	6.56±1.33	1.70±1.11	5.55±0.83	3.64±1.97
	Quartile 2	144.9±23.6	26.1±4.2	6.59±1.32	1.70±1.01	5.53±0.67	3.71±1.95
	Quartile 3	145.0±23.7	25.9±4.3	6.63±1.33	1.68±1.10	5.53±0.71	3.68±1.74
	Quartile 4	144.9±23.7	25.9±4.1	6.58±1.31	1.72±1.15	5.53±0.69	3.78±1.91
rs2298850	Major homozygote	145.3±24.0	26.0±4.2	6.58±1.33	1.70±1.08	5.55±0.75	3.73±1.99
	Heterozygote	144.6±23.6	25.9±4.1	6.60±1.32	1.68±1.09	5.51±0.69	3.65±1.72
	Minor homozygote†	146.0±23.9	25.9±3.9	6.57±1.32	1.75±1.17	5.51±0.63	3.67±1.87
rs10741657	Major homozygote†	144.3±23.3	25.9±4.1	6.57±1.34	1.70±1.12	5.53±0.68	3.76±1.96
	Heterozygote	145.7±23.9	26.1±4.2	6.60±1.31	1.71±1.09	5.53±0.73	3.66±1.83
	Minor homozygote	144.9±24.5	25.9±4.2	6.58±1.32	1.65±1.02	5.54±0.79	3.69±1.93
rs3794060	Major homozygote	144.7±23.8	26.0±4.2	6.56±1.32	1.67±1.06	5.56±0.83	3.63±1.76
	Heterozygote	144.9±23.5	26.0±4.1	6.59±1.32	1.72±1.11	5.52±0.67	3.74±2.00
	Minor homozygote†	146.5±24.8	26.0±4.2	6.62±1.33	1.71±1.12	5.52±0.60	3.79±1.88
rs6013897	Major homozygote	145.0±24.0	25.9±4.1	6.60±1.31	1.72±1.10	5.52±0.71	3.56±1.87*
	Heterozygote	145.2±23.5	26.0±4.2	6.57±1.34	1.67±1.09	5.54±0.75	3.88±1.96
	Minor homozygote†	145.2±23.7	26.1±4.3	6.59±1.27	1.73±1.04	5.57±0.71	3.98±1.79

* P<0.05 for linear trend across genotype, linear regression with gender, age, BMI, vitamin D/cod liver oil supplementation and season as covariates. Correction for multiple comparisons for the individual SNPs was performed with a factor of 5.

** Analyzed in 4613 subjects;

*** Analyzed in 3809 subjects in the fifth survey in 2001–2002.

**** Quartile with highest serum 25(OH)D level.

† Risk allele.

### Major endpoints

**Figure 1:**
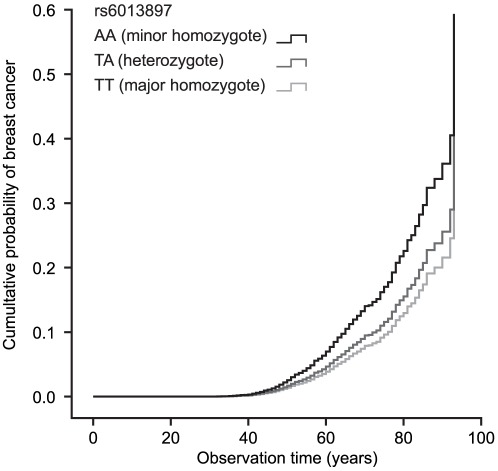
Cumultative probability of first breast cancer according to rs6013897 genotypes based on Cox regression with age and BMI as covariates.

Neither the genotype score nor any of the four selected SNPs were significantly associated with MI, T2DM, cancer and mortality after correction for multiple testing (Table 4). For MI inclusion of the risk factors systolic blood pressure and serum cholesterol did not affect these results (data not shown). When cancer was subdivided according to location (breast, lung, colorectal and prostate), rs6013897 at the *CYP24A1* gene showed a significant relation with breast cancer (P<0.05) where the minor homozygote, which was the one with the lowest serum 25(OH)D level, had an 86% higher risk of developing breast cancer than the major and reference homozygote (Figure 1). This was the only one of the four selected SNPs, as well as the genotype score, that showed a significant relation to a specific cancer.

### CVD risk factors

There was no significant relation between CVD risk factors and the genotype score or the four selected SNPs (Table 6). There was no difference in smoking habits in relation to any of the SNP genotypes (data not shown).

## Discussion

To our knowledge, this is the first large population based study where SNPs associated with the serum 25(OH)D levels have been reported in relation to the major diseases CVD, T2DM, cancer, mortality, and risk factors for disease. As expected we found a significant relation between the selected SNPs and serum 25(OH)D levels [13], [14], whereas the relations with the hard endpoints and CVD risk factors were not consistent.

The SNPs analysed by us are all involved in important steps in vitamin D metabolism like availability of substrate for vitamin D production in the skin (*NADSYN1* gene), hydroxylation to 25(OH)D (*CYP2R1* gene), transport of 25(OH)D in the circulation (*DBP* or *GC* gene) and degradation to inactive metabolites (*CYP24A1* gene) [13]. The SNPs are not influenced by life-style, and their effects are life-long. Accordingly, they may potentially be better markers of a subject's vitamin D status than a single serum 25(OH)D measurement. Although no substitute for RCTs, the identification of these SNPs has made it possible to indirectly test the relation between serum 25(OH)D and diseases. In particular, one could expect to see a relation with diseases that develop over a long period of time like CVD, T2DM, cancer and ultimately also death.

Regarding mortality, where a low serum 25(OH)D level is a predictor [7], [9], all hazard ratios were close to 1.00 with no trend for the low genotype score, nor the individual genotypes associated with high serum 25(OH)D levels, to have a protective effect. Similarly, low serum 25(OH)D is a predictor of MI [29], but none of the genotypes for the SNPs examined appeared related to MI risk. This was not changed by inclusion of the risk factors systolic blood pressure and serum cholesterol in the Cox regression analyses. Although not statistically significant, it was also noteworthy that for rs2298850 (which was the SNP with the highest difference between major and minor homozygotes regarding serum 25(OH)D)) there was 17% reduced risk of MI for the genotype with the lowest serum 25(OH)D level. An association between SNPs in the *CYP24A1* gene and MI could have been expected as Sehn et al. [24] reported one such SNP (rs2762939) to be related to coronary artery calcification, which again predicts CVD [30]. However, none of the *CYP24A1* gene SNPs, (including rs2762939) appeared related to MI in our cohort.

A relation between vitamin D and cancer is plausible as VDR, which is a nuclear receptor, is found in cells in a number of tissues and the active form of vitamin D, 1,25-dihydroxyvitamin D (1,25(OH)_2_D), appears to have anti-proliferative effects [31]. Epidemiological data indicate a negative prognostic role for low serum 25(OH)D levels regarding cancer [32], but so far a cause-and-effect relationship has not been established [33]. In line with this, for the genotype score quartiles and most of the SNPs analyzed by us the hazard ratios for cancer were close to 1.00 except for rs6013897. For this SNP the genotype associated with the lowest serum 25(OH)D level had a 20% increased risk of cancer, but this was not significant after adjustment for multiple comparisons.

As our cohort included as many as 2924 cases with cancer, we also investigated breast, colorectal, lung and prostate cancer separately. For lung cancer [34] as well as for prostate cancer [35] there are only weak indications for a relation with vitamin D, and neither for the genotype score quartiles nor the SNPs analyzed by us were there any significant associations. For breast cancer, the situation is similar with no consensus on whether serum 25(OH)D is predictive of future disease or not [36]. However, for the SNP rs6013897, subjects with the minor homozygote genotype (which was the one with the lowest serum 25(OH)D level), had almost twice the risk of developing breast cancer as compared to subjects with the major homozygote genotype.

The rs6013897 is related to the *CYP24A1* gene which is considered as an oncogene. This gene (the 24-hydroxylase enzyme) converts 25(OH)D as well as 1,25(OH)_2_D to their inactive forms and is present also in peripheral tissues. Locally produced 1,25(OH)_2_D is assumed to have an anti-cancer effect, and an increased local expression of the *CYP24A1* gene (and thereby increased degradation of 1,25(OH)_2_D) has been associated with poor survival [37]. It is therefore reasonable that also polymorphisms in this gene may affect risk of cancer. However, Anderson et al. [38] who examined 1560 subjects with breast cancer and 1633 controls found no relation between SNPs in *CYP24A1* gene and breast cancer, whereas they reported an association between breast cancer and rs7041 which was not seen in our study (data not shown). And to add to the confusion, in a study from China including 2919 cases and 2323 controls, neither rs7041 nor rs2762932 (proxy for rs6013897) were considered significantly related to breast cancer [39].

For colorectal cancer there appears to be an inverse relation with intake of vitamin D as well as with serum 25(OH)D levels [40]. However, RCT evidence is limited, and none of the SNPs examined by us, or the genotype score, showed a significant association with colorectal cancer. Similar to the other endpoints, there was no consistent trend for a protective effect of genotypes associated with high serum 25(OH)D levels.

The cross-sectional associations between serum 25(OH)D levels and risk factors for disease like blood pressure, BMI, serum lipids and HbA_1c_ are undisputable. From our own studies we have found the unadjusted mean difference between the lower and higher quartiles of serum 25(OH)D to be 7.7 mmHg for systolic blood pressure, 1.4 kg/m^2^ for BMI, 0.08% for HbA_1c_ and 0.37 mmol/L for TG [2]–[5]. These relations with risk factors could possibly explain the association between serum 25(OH)D levels and CVD [29], T2DM [6], [8] and mortality [9]. However, after correction for multiple testing no significant findings regarding SNPs and CVD risk factors were found. On the other hand, we did find a significant relation for rs6013897 where the genotype with the highest serum 25(OH)D level had the lowest serum PTH level, indicating at least some physiological importance for that SNPs in calcium metabolism.

There could be a number of reasons why we were not able to disclose the presumed relations between the SNPs and health. First of all, the mean differences between the genotypes with the highest and lowest serum 25(OH)D levels, and also between the first and fourth genotype score quartiles, were no more than 8 nmol/L. This was substantially lower than that reported by Wang et al. who found differences of 18 nmol/L in the Framingham Heart Study and 13 nmol/L in the 1958 British Birth Cohort [13]. In the power calculations we therefore assumed a greater difference in serum 25(OH)D levels than what we actually found. We also made the calculations based on cross-sectional and longitudinal associations between serum 25(OH)D levels and diseases which may not reflect causal relations. The power calculation may therefore have been too optimistic, and the study underpowered. Secondly, for SNPs at the *NADSYN1* gene the population was not in Hardy-Weinberg equilibrium and the results for that gene may therefore have been biased. This could possibly be explained by the high proportion of the Tromsø population having Sami ancestry [41] as SNPs in the *NADSYN1* gene show a remarkable difference in allele frequency at least between Asian and Western populations [42]. Unfortunately, we did not have any ancestry-informative SNPs that could verify this hypothesis.

Furthermore, the SNPs with the greatest difference between genotypes were related to the *DBP* gene. These same polymorphisms are strongly related to the concentration of DBP [13], and the serum 25(OH)D assays used today measure total 25(OH)D and not only the free fraction. Presumably it is the free fraction of 25(OH)D that is the biologically active, and changes in the total amount of 25(OH)D induced by changes in the DBP concentration might therefore be misleading. Also, if the SNP-induced changes in amount of DBP are accompanied by changes in affinity between binding protein and 25(OH)D, the differences in total 25(OH)D will be even more difficult to interpret [43]. In addition, the differences between the genotypes for the other SNP were rather small, and no greater than 3 nmol/L. Accordingly, it would for these SNPs be very difficult to disclose an effect on hard endpoints as well as risk factors. And finally, one may speculate that an effect of small differences in serum 25(OH)D, as those induced by the SNPs in our study, would be most evident in a vitamin D depleted population. Although Tromsø is located far North (at 69°N), and the average serum 25(OH)D levels not optimal, it was still not much different from what is seen in the rest of Western Europe [29], and our results may therefore not apply in areas of profound vitamin D deficiency.

The classical effect of vitamin D is increased intestinal calcium absorption, and vitamin D deficiency leads to rickets in children and osteomalacia and/or osteopenia in adults [1]. These relations are well documented and it might be easier to document an effect of vitamin D SNPs on the skeleton than on MI, T2DM, cancer and mortality. It should also be noted that even if a consistent relation between SNP genotypes and disease had been found, that would only have been an indication of causality and no proof. Thus, the SNPs could very well be associated with other genes that were the ones truly causing the observed effect, and the vitamin D SNPs therefore only markers of disease risk.

We analysed 17 SNPs known to be associated with the serum 25(OH)D level. For each of the four related genes we selected the SNP with the highest difference in serum 25(OH)D between the genotypes. Furthermore, we analyzed four hard endpoints and also subdivided one of them, cancer, according to organ location. Accordingly, we had to correct for multiple comparisons and the P-values for the hard endpoints were corrected with the Bonferroni method. However, even if we had used a less conservative approach like correcting with a factor of four (because of the number of SNPs pre-selected for analyzes), no additional relations with the hard endpoints would have reached statistical significance. Furthermore, in the principal analysis we used a combined genotype score without correction of the P-value, and still no significant associations with the end points were seen.

It must also be emphasised that our main hypothesis was that genotypes associated with high serum 25(OH)D level would “protect” against disease. However, we found no such trend. In this context it is worth mentioning that although the literature on SNPs affecting the serum 25(OH)D level and health is sparse, there is an abundance of such publications for SNPs in the *VDR* gene [44]. These publications have shown very little consistency, which may reflect that for normal biological effects of vitamin D, polymorphisms characterised so far are of limited importance. On the other hand, a significant relation between SNPs in the *CYP2R1*, *DHCR7* (*NADSYN1*) and *CYP27B1* genes and predisposition to type 1 diabetes have been reported [45], and genetic polymorphisms in the vitamin D system may be more important in the pathogenesis of autoimmune diseases than in the diseases studied by us.

In addition to the number of shortcomings, our study also has some strengths. The endpoint registers were carefully established and quality checked, the number of subjects with endpoints was substantial, and the study was population based. The case-cohort study design used by us is also well suited if more than one endpoint is to be evaluated [19].

In conclusion, we have not been able to document consistent relations between SNPs associated with serum 25(OH)D levels and MI, T2DM, cancer, mortality or CVD risk factors, with a possible exception for rs6013897 and breast cancer. This does not exclude that these SNPs contribute in the pathogenesis of MI, T2DM or cancer, but makes it unlikely that they have a major influence. Further studies are needed to evaluate these SNPs in relation to other diseases, in populations with different genetic background, as well as in vitamin D depleted populations.
